# The known genetic loci for telomere length may be involved in the modification of telomeres length after birth

**DOI:** 10.1038/srep38729

**Published:** 2016-12-08

**Authors:** Qiao Weng, Jiangbo Du, Fei Yu, Tongtong Huang, Mengxi Chen, Hong Lv, Hongxia Ma, Zhibin Hu, Guangfu Jin, Yali Hu, Hongbing Shen

**Affiliations:** 1 Drum Tower Clinical Medical College, Nanjing Medical University, Nanjing 210008, China; 2 State Key Laboratory of Reproductive Medicine, Nanjing Medical University, Nanjing 211166, China; 3 Department of Epidemiology and Biostatistics, School of Public Health, Nanjing Medical University, Nanjing 211166, China; 4 Department of Obstetrics and Gynecology, Nanjing Drum Tower Hospital, Affiliated to Nanjing University Medical School, Nanjing 210008, China

## Abstract

Telomere length varies considerably among individuals. It is highly heritable and decreases with ageing or ageing related diseases. Recently, genome-wide association studies (GWAS) have identified several genetic loci associated with telomere length in adults. However, it is unclear whether these loci represent the genetic basis of telomere length or determine the individual susceptibility to shortening during growth process. Using DNA extracted from peripheral and cord blood of 444 mother-newborn pairs from a Chinese population, we measured relative telomere length (RTL) and genotyped eight known telomere length related variants that were initially identified in populations of European descent. We observed the T allele of rs10936599 and the T allele of rs2736100 were norminally associated with shorter RTL (*P* = 0.041 and 0.046, respectively) in maternal samples. Furthermore, the Weighted genetic score (WGS) of eight variants was significantly associated with RTL in maternal samples (R^2^ = 0.012, *P* = 0.025). However, we didn’t detect any significant associations for any individual variant or the combined WGS with RTL in newborns. These findings didn’t support the hypothesis that telomere length related loci may affect telomere length at birth, and we suggested that these loci may play a role in telomere length modification during life course.

Telomeres are tandem repeats of TTAGGG nucleotides at the ends of eukaryotic chromosomes and crucial in protecting the chromosomes from deterioration and rearrangement^1^. During normal somatic cell divisions in human, telomeres are progressively attrited due to the ‘problem of linear chromosome’ (incomplete replication at 3′ end of chromosomes)^2^, eventually reaching a critical length that leads to cell senescence^3^. Telomeres can be lengthened usually by the activation of telomerase reverse transcriptase (TERT)^4^ or rarely through the ALT (alternative lengthening of telomeres) pathway^5^. Telomere length has been extensively implicated with the risk of cardiovascular disease^6^, metabolic disease^7^, chronic obstructive pulmonary disease (COPD)^8^,^9^, malignant tumor^10^, and infection^11^. Therefore, telomere shortening is a biomarker for biologic aging as well as development and progression of disease^12^.

Telomere length exhibits considerable inter-individual variability. Intra-uterine variables including genetic and other factors during pregnancy determine the telomere length of individuals at birth, and external environmental factors advance or slow down the attrition of telomere after birth^13^. Genetic determinants of telomere length have been widely investigated. Twins studies indicate that heritable factors may contribute up to 80% of the inter-individual variation of telomere length^14^,^15^. Quantitative trait linkage studies have mapped several loci for telomere length^16^,^17^,^18^. Recently, genetic variants at chromosomes 2p16.2 (*ACYP2*), 3q26 (*TERC*), 5p15.33 (*TERT*), 4q32.2 (*NAF1*), 10q24.33 (*OBFC1*), 19p12 (*ZNF208*) and 20q13.3 (*RTEL1*) have been identified to be associated with telomere length in three genome-wide association studies (GWAS) of European descent^19^,^20^,^21^,^22^. Thereafter, the association of 5p15.33 (TERT) with telomere length was also replicated in Chinese populations^23^,^24^. However, all of these studies evaluated the association between genetic variants and telomere length in adult subjects, which represents both telomere length at birth and telomere attrition after birth. Therefore, it is unclear whether these identified loci directly determine the telomere length in the foetal period or influence the shortening of telomere in growth process.

Herein, we designed a study including 444 mother-newborn pairs in a Chinese population, to compare the associations of the identified loci with telomere length between maternal peripheral blood and cord blood of newborns.

## Results

**Figure 1:**
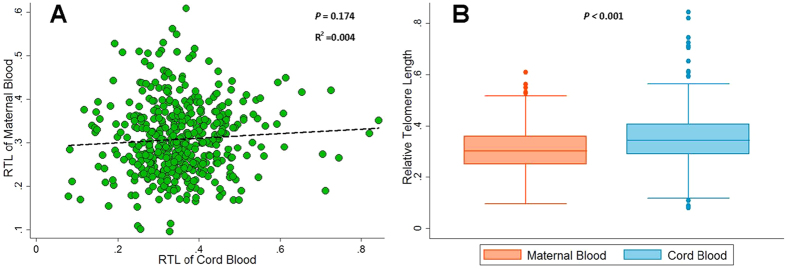
(**A**) Correlation between relative telomere length (RTL) of maternal blood and cord blood (R^2^ = 0.004, *P* = 0.174); (**B**) Comparison of relative telomere length (RTL) means between maternal blood and cord blood (*P* < 0.001).

A total of 444 pregnant women were enrolled into our study. Table 1 shows the characteristics of pregnant women and newborns. The age at current pregnancy of women were ranging from 19 to 42 years, and 80.86% were in the 25~35 age group. Few children were born before 37 weeks of gestation (n = 45, 10.14%) or with a birth weight < 2,500 g (n = 35, 7.88%) (Table 1). No statistically significant correlation was observed between RTL of maternal and cord blood (Fig. 1A). The overall RTL of maternal blood was significantly shorter (mean: 0.309) than of cord blood (mean: 0.354, *P* < 0.001), and this consistent trend was observed in all subgroups although not all the subgroups reached the statistically significant level (Fig. 1B, Table 1).

Genotyping call rates of all the selected genetic variants were higher than 95%, and the observed genotype frequencies for these variants were in Hardy-Weinberg equilibrium (*P* > 0.00625) among both the maternal or cord blood samples (Supplementary Table 1). We evaluated the association between genetic variants and telomere length among mother or newborns. In maternal samples, we found that the T allele of rs10936599 and the T allele of rs2736100 were norminally associated with short RTL (*P* = 0.041 and 0.046, respectively), but none of the variants were significant after Bonferroni correction. Both of the associations were consistent with that reported in populations of European descent. No significant associations were observed between the other genetic variants and RTL in maternal samples. However, we did not find significant association between the genotypes of these eight genetic variants and RTL in cord blood (Table 2).

**Figure 2:**
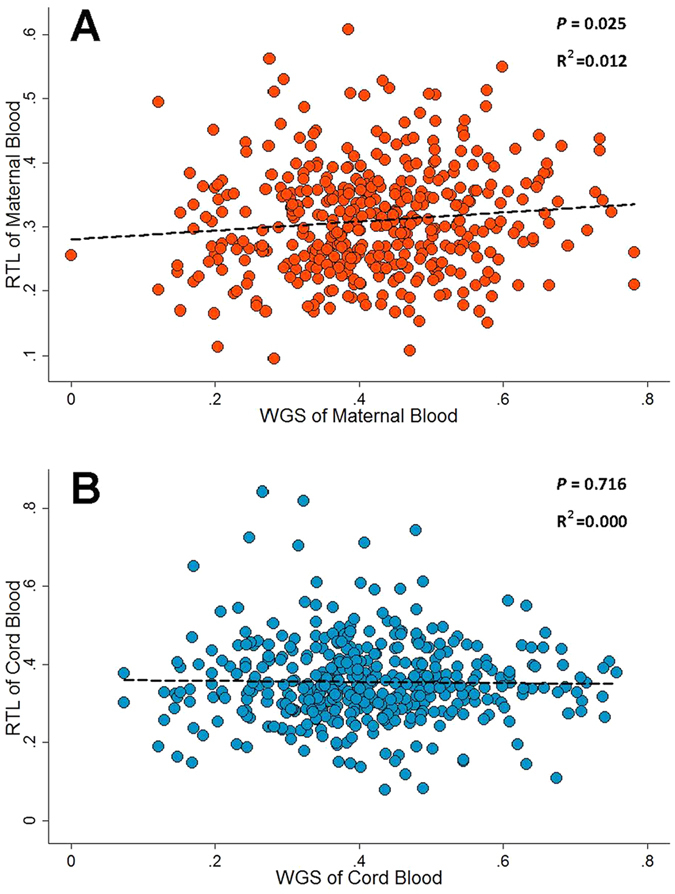
Correlation between relative telomere length (RTL) and weighted genetic score (WGS) of eight genetic variants. (**A)** RTL of maternal blood *vs* WGS of maternal blood; (**B)** RTL of cord blood *vs* WGS of cord blood).

We further calculated WGS to analyze the cumulative effect of eight loci on telomere length. As expected, we observed significant correlation between maternal WGS and RTL (R^2^ = 0.012, *P* = 0.025, Fig. 2A). However, there were no association between cord blood WGS and RTL (R^2^ = 0.000, *P* = 0.716, Fig. 2B).

## Discussion

In our study, we observed that rs10936599 and rs2736100 related to telomere length in Europeans were also associated with maternal telomere length in Chinese. Rs10936599 and rs2736100 were located in *TERC* (telomerase RNA component) locus on 3q26 and *TERT* (telomerase reverse transcriptase) locus on 5p15, respectively. *TERC* encode the RNA component, and *TERT* encode the catalytic subunit of telomerase reverse transcriptase, both the two genes are key components of telomerase. Although the other 6 variants were not significantly associated with telomere length of maternal, 4 of these variants were in the same association direction with those observed in populations of European descent. The sample size may be the main limitation to fully detect the modest effect of individual variant on telomere length.

In previous study, genetic variants associated with telomere length were discovered and replicated in adults, which, however, was believed to explain a fraction of heritability of telomere length. If it is true, we supposed that the association between genetic variants and RTL in cord blood may be stronger than that in maternal blood, because the confounding factors among postnatal exposure can be reduced by using the cord blood sample to detect the genetic association. However, it is out of our expectation that we did not observe any association between genetic variants and RTL in cord blood either in the single variant analysis or WGS analysis. These findings suggest that the known telomere length related variants may not directly affect the telomere length in the foetal period or at birth of individuals, but influence the maintaining of telomere homeostasis or the resistance to the risky external stimulus in the growth process of individuals.

The hereditability of telomere length has been firstly proved in a twin study about twenty years ago^14^. However, the mode of its inheritance was still unclear. A terminal restriction fragment (TRF) based study has firstly proposed that X-linked inheritance of telomere length is a probable genetic pattern^25^, because significant associations of telomere length were observed between mother-daughter, mother-son, and father-daughter, but not between father-son. In contrast, a positive linkage between paternal age and telomere length of offspring was revealed in another study^26^. More interestingly, two following studies reported a significant association between fathers and offspring but no significant association between mothers and children^27^,^28^, suggesting a paternal inheritance mode of telomere length. In the current study, we did not find a significant association between telomere length of maternal blood and cord blood, which was in accordance with the previous findings and confirmed the potential paternal inheritance pattern telomere length. All of these evidences point to a gene imprinting mechanism in telomere length regulation, rather than the X chromosome genetic inheritance.

In our study, we found that RTL of cord blood was 15% longer than that of maternal blood. But we did not observe a reverse association between age and RTL in pregnant women. One of the possible explanations is the narrow age span of our participants. The range of age at current pregnancy in our study is 19 to 42 and the majority was 25 to 35 years old. Moreover, there were mounting evidences indicating that gradual loss of telomeric repeat sequences with aging is not linear; the velocity of telomere attrition is more rapid during childhood and adolescence, remaining relatively stable in adulthood, and thereafter is followed by a gradual loss of telomere repeats at old age^3^,^25^,^26^,^29^,^30^. In addition, only 32 pregnant women were older than 35, so the small sample size in this subgroup may cause some deviation as the data might be not stable enough. This reason may lead to the unexpected phenomenon. The above reasons may dilute the adverse effects of ageing on telomere length in our study.

Many studies have reported that telomere length in adult women were significantly longer than that in men^16^,^23^,^25^,^31^,^32^,^33^. Recently, Benetos *et al*. indicated that the sex difference in telomere length is largely determined in utero, based on the study of telomere length dynamics in adult same-sex twins and opposite-sex twins. This intra-uterine effect was attributed to the intrauterine sex hormonal environment^13^. In our study, although we observed that the RTL in female newborns was 4% longer than that in male newborns, but there was no significant difference. It could be due to the limited sample size. Therefore, the potential effect of the intra-uterine environment on the sex difference in telomere length is an interesting and noteworthy issue.

In our study, no difference was found in newborn RTL as a function of maternal age, birth weight, and gestational age in our study. Part of our findings can be supported by the following evidences. De Meyer *et al*. found that paternal age was a vital determinant for newborn RTL while maternal age was not independently related to newborn RTL^34^. Researchers also have indicated that there were no difference between the RTL of preterm neonates and full-term newborns^35^. In addition, several studies observed that higher birth weight was associated with longer RTL^36^,^37^, but which were inconsistent with our findings. Possible reason is that intra-uterine variables that affect newborn RTL are extremely complex, maternal psychosocial stress^38^, maternal estriol concentrations^39^, maternal Folate Concentrations^40^ and undetected factors might play an important role in affecting newborn RTL and disturbed the correlation between birth weight and RTL. Besides, it is the first time that the relationship between newborn RTL and mode of delivery had been evaluated and there seemed to be no significant association in our current study. However, more well-designed studies are still needed to explore the relationship between maternal conditions and newborn RTL.

The advantages and limitations of the current study should be addressed. The major advantage is that we systematically investigated the association between telomere length and genetic variants in mother-newborn pairs and this design may help to clarify inheritance mechanism of telomere length. Nevertheless, there still existed a lot of deficiencies. Firstly, we did not get the blood samples of the husbands of the pregnant women. So we could not further evaluate the association between RTL of father-newborn pairs, which is crucial for the clearly illustrating of the inheritance pattern of telomere length. Secondly, our current analysis lack of the information about maternal lifestyle (smoking status, alcohol consumption, physical activity, etc) and maternal health condition (obesity, hypertension, etc), which were very important and should be taken into our further study. At last, we used the “*Power and Sample Size Calculation software*” to evaluate statistic power. We controlled type I error rate to be 0.05 and other relevant parameters derived from our current data: slope of the line obtained by regressing RTL against genotype ranges from 0.0002 to 0.012, the standard deviation of the regression errors ranges from 0.082 to 0.1 and the standard deviation of genotype ranges from 0.51 to 0.74. As a result, the statistic power ranges from 5% to 54% based on 444 participants. Statistical power might be improved with larger sample size, while the limited sample size might constrain our capacity to find the positive correlation. Therefore, further studies based on trio samples with large sample size may facilitate to evaluate our findings.

In summary, our study did not support the hypothesis that telomere length related loci identified in European may affect telomere length at birth in Chinese, and we suggested that these loci may play an important role in telomere length modification during life course.

## Materials and Methods

### Study participants

Subjects of current study were enrolled from Nanjing Drum Tower Hospital in Nanjing, Jiangsu province of eastern China. During the recruitment period from April 2014 to April 2015, a total 444 singleton pregnant women were included in our study. Detailed information on maternal characteristics, birth weight, gestational age, infant sex, and mode of delivery was obtained from maternity records. Gestational age (completed weeks) was calculated based on last menstrual period or ultrasound-based estimated date of conception. Venous blood samples of mother were collected from 444 pregnant women before or during the delivery period. Paired umbilical cord blood samples were collected immediately after birth from the cord vein of newborns and locally stored at −20 °C. Samples were then shipped on dry ice to the study laboratories and genomic DNA was extracted from peripheral blood of mothers and cord vein blood of newborns. This study was approved by the ethics committees of Nanjing Medical University and Nanjing Drum Tower Hospitals and all experiments were performed in accordance with relevant guidelines and regulations. Written informed consent was obtained from all participating women.

### Measurement of relative telomere length

Based on a modified quantitative polymerase chain reaction (qPCR) protocol^23^, we measured telomere length using ABI PRISM 7900HT Sequence Detection System (Applied Biosystems). Firstly, the reference DNA (pooled from 5 adults’ samples) was used to draw a standard curve with concentrations ranging from 0.25 to 8 ng/μL, and linear correlation between input DNAs and Ct value (r^2^ > 0.99) was observed over this range. We computed the ratio of telomere repeat copy number (T) between a single-gene copy number (S) to reflected the relative telomere length (RTL), and reported as individual sample T/S ratio corrected for the reference DNA. The equal slope of standard curves indicated the equal amplification efficiencies between the telomeric sequence and single-copy gene sequence. Samples were rerun at a suitable concentration to make sure that they were amplified within the linear range if their threshold cycle (Ct) numbers fell outside the scope defined by the standard curves. The primers sequences for telomere and single-copy gene (*36B4*) amplification were as follow: TEL1, 5′-GGTTTTTGAGGGTGAGGGTGAGGGTGAGGGTG AGGGT-3′; TEL2, 5′-TCCCGACTATCCCTATCCCTATCCCTATCCCTATC CCTA-3′; 36B4u, 5′-CAGCAAGTGGGAAGGTGTAATCC-3′; and 36B4d, 5′-CCCATTCTATCATCAACGGGTACAA-3′. The same reference DNA was adopted in all runs to control the inter-plate variation. Each reaction well contained 10 μl SYBR® Green PCR Master Mix (Applied Biosystems) and the final DNA concentration of 5 ng/μl. All samples were assayed in duplicate wells and we use the average values of two measurements in the statistical analyses. Equal maternal and cord blood DNA samples were assayed on each reaction plate, and technicians were blinded to the grouping status. RTL was calculated based on Cawthon’s formula^41^


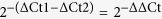


### Genetic variants selecting and genotyping

The known telomere length related variants were filtered on the basis of the following criteria: (1) the reported significance level of association reaching 5 × 10^−8^; (2) the minor allele frequency (MAF) of variants not less than 5% in Chinese population; (3) for those variants that were in linkage disequilibrium (LD) at r^2^ > 0.5, only one variant will be selected. The MAF and LD data were obtained from the 1000 Genomes Project CHB + JPT subjects (Phase I interim release). As a result, we selected seven telomere length related variants from three GWASs^19^,^20^,^22^, including rs10936599 (3q26.2, *TERC*), rs11125529 (2p16.2, *ACYP2*), rs2736100 (5p15.33, *TERT*), rs4387287 (10q24.33, *OBFC1*) rs755017 (20q13.33, *RTEL1*), rs7675998 (4q32.2, *NAF1*), and rs8105767 (19p12, *ZNF208*). Besides, we also included rs2736108 at 5p15.33 (*TERT*), which was related to telomere length in a GWAS^22^ though the reported *P* value did not reach genome-wide significant level (only 5.8 × 10^−7^). Therefore, we finally selected eight variants to be genotyped in current study.

We performed genotyping by using the TaqMan allelic discrimination assay on the ABI PRISM 7900HT Sequence Detection System. Details of the primers and probes were displayed in our previous study^23^. The quality control procedures in genotyping assay are as follow: (1) Two negative controls were included in each 384-plate, and equal maternal and cord DNA samples were assayed on each 384-plate, all the technicians were blinded to the grouping status; (2) 5% samples were run in duplicate in order to evaluate the concordance rate, those samples with different genotyping results should be re-detected; (3) Only those variants with call rate higher than 95% can be selected in further analysis. The genotyping results were determined by using the SDS 2.3 Allelic Discrimination Software (Applied Biosystems).

### Calculation of genetic scores

In order to assess the combined effect of the eight telomere length related genetic variants, Weighted genetic score (WGS)^42^ was calculated based on the genotypes of those variants. For each individual, WGS was calculated by multiplying the number of risk alleles by the telomere length associated beta (*β*_*j*_), which was derived from the regression analysis of telomere length and genotype in the current study. For each SNP, we appointed the longer telomere length related allele as risk allele. To calculate WGS for the i-th subject, the following formula was used:


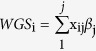


In this formula, *x*_*ij*_ is the number risk alleles for the *j*-th variant in the *i*-th subject (*x*_*ij*_ = 0, 1 or 2) and *β*_*j*_ is the coefficient or weight for the *j*-th variant.

### Statistical analysis

RTL from our data was similar to normal distribution. Paired t-test was used to compare the differences between mother-newborn pairs for RTL. One-way anova or t-test was employed to examine the differences of RTL between subgroups divided by selected characteristics. Generalized linear models (GLMs) were used to conduct tests for correlation between RTL and genotype of genetic variants, WGS, and other continuous variables. Bonferroni correction was used for multiple testing during the genetic association analysis and Hardy-Weinberg equilibrium analysis and the significance level was defined at 0.00625 (0.05/8 tests). A *P*-value less than 0.05 was considered statistically significant unless specifically notified. General analyses were performed with Stata version 9.2 (StataCorp LP).

## Additional Information

**How to cite this article**: Weng, Q. *et al*. The known genetic loci for telomere length may be involved in the modification of telomeres length after birth. *Sci. Rep.* **6**, 38729; doi: 10.1038/srep38729 (2016).

**Publisher's note:** Springer Nature remains neutral with regard to jurisdictional claims in published maps and institutional affiliations.

## Supplementary Material

Supplementary Dataset 1

## Figures and Tables

**Table 1:** Selected characteristics of pregnant women and newborns and distributions of relative telomere length (RTL) between maternal blood and cord blood.

**Variables**	No. (%)	RTL of maternal blood	RTL of cord blood	*P*^a^
Age at current pregnancy (Years)				
<25	53(11.94)	0.320 ± 0.091	0.358 ± 0.095	0.052
25~35	359(80.86)	0.305 ± 0.080	0.354 ± 0.106	0.000
≥35	32(7.21)	0.327 ± 0.105	0.343 ± 0.087	0.534
*P*^b^		0.042	0.260	
Season of delivery				
March–May	153(34.46)	0.309 ± 0.077	0.358 ± 0.102	0.000
June–August	165(37.16)	0.315 ± 0.087	0.353 ± 0.118	0.001
September–November	93(20.95)	0.296 ± 0.080	0.350 ± 0.081	0.000
December–February	33(7.43)	0.314 ± 0.097	0.350 ± 0.086	0.124
*P*^b^		0.350	0.947	
Gestational age (weeks)				
<37	45(10.14)	0.310 ± 0.074	0.369 ± 0.119	0.010
≥37	399(89.86)	0.308 ± 0.084	0.352 ± 0.101	0.000
*P*^b^		0.897	0.294	
Birth weight (g)				
<2,500	35(7.88)	0.318 ± 0.090	0.372 ± 0.135	0.069
≥2500	409(92.12)	0.308 ± 0.083	0.352 ± 0.100	0.000
*P*^b^		0.492	0.281	
Mode of delivery				
Vaginal	363(81.76)	0.309 ± 0.085	0.353 ± 0.102	0.000
Cesarean section	81(18.24)	0.308 ± 0.078	0.358 ± 0.112	0.002
*P*^b^		0.905	0.685	
Sex of newborns				
Boy	226(50.90)	0.306 ± 0.083	0.347 ± 0.103	0.000
Girl	218(49.10)	0.311 ± 0.083	0.361 ± 0.104	0.000
*P*^b^		0.579	0.133	

^a^*P* value for paired t-test that was used to compare the differences between mother-newborn pairs for RTL. ^b^*P* value for one-way anova or t-test that was employed to examine the differences of RTL between subgroups divided by selected characteristics.

**Table 2:** Regression analysis of reported telomere length related loci with relative telomere length (RTL) among pregnant women and newborn.

Locus [reference]	Chr.	Associated gene	Reported effect allele^a^	Alternative allele	Maternal genotype and RTL^b^			Cord genotype and RTL^b^		
					EAF^c^	β	*P*	EAF	β	*P*
rs10936599^19^	3q26.2	*TERC*	T	C	0.574	−0.0119	0.041	0.566	−0.0017	0.819
rs11125529^19^	2p16.2	*ACYP2*	C	A	0.811	0.0019	0.792	0.825	−0.0012	0.892
rs2736100^19^	5p15.33	*TERT*	T	G	0.570	−0.0110	0.046	0.594	0.0052	0.470
rs2736108^22^	5p15.33	*TERT*	G	A	0.684	−0.0080	0.176	0.722	−0.0020	0.799
rs4387287^21^	10q24.33	*OBFC1*	C	A	0.840	−0.0027	0.725	0.847	−0.0010	0.920
rs755017^19^	20q13.33	*RTEL1*	A	G	0.590	−0.0017	0.779	0.573	0.0023	0.736
rs7675998^19^	4q32.2	*NAF1*	A	G	0.155	−0.0054	0.493	0.159	−0.0005	0.958
rs8105767^19^	19p12	*ZNF208*	A	G	0.711	0.0061	0.307	0.719	−0.0002	0.978

^a^The reported effect allele associated with short telomeres in populations of European descent.

^b^Derived from generalized linear models. The reference homozygotes, heterozygotes and effect homozygotes were encoded as 0, 1 and 2, respectively.

^c^EAF: Effective allele frequency.
