# Supplementary material for: The known genetic loci for telomere length may be involved in the modification of telomeres length after birth
Source: Sci Rep. 2016 Dec 8;6:38729. doi: 10.1038/srep38729 (PMC5143977; doi:10.1038/srep38729)
Supplement: Supplementary Dataset 1 [file srep38729-s1.docx]

**The known genetic loci for telomere length may be involved in the modification of telomeres length after birth**

Qiao Weng ^1,2,4†^, Jiangbo Du ^2,3†^, Fei Yu ^2,3†^, Tongtong Huang ^3^, Mengxi Chen ^3^, Hong Lv ^3^, Hongxia Ma ^3^, Zhibin Hu ^2,3^, Guangfu Jin ^2,3*^, Yali Hu ^1,4*^, Hongbing Shen ^2,3*^

**Supplementary Table 1.** Details about genotype of the selected SNPs in current study

| SNP | Call rate^a^ | Call rate^b^ | Effect allele | EAF^a^ | EAF^b^ | *P* for HWE *^a^* | *P* for HWE *^b^* |
| --- | --- | --- | --- | --- | --- | --- | --- |
| rs10936599 | 99.55% | 98.65% | T | 0.57 | 0.57 | 0.33 | 0.03 |
| rs11125529 | 97.30% | 98.65% | C | 0.81 | 0.83 | 0.75 | 0.87 |
| rs2736100 | 100.00% | 97.75% | T | 0.57 | 0.59 | 0.38 | 0.84 |
| rs2736108 | 99.55% | 97.97% | G | 0.68 | 0.72 | 0.51 | 0.47 |
| rs4387287 | 98.65% | 98.20% | C | 0.84 | 0.85 | 0.86 | 1.00 |
| rs755017 | 99.77% | 98.42% | A | 0.59 | 0.57 | 0.01 | 0.01 |
| rs7675998 | 99.32% | 98.42% | A | 0.16 | 0.16 | 0.72 | 1.00 |
| rs8105767 | 99.32% | 96.62% | A | 0.71 | 0.72 | 0.25 | 0.17 |

EAF: effect allele frequency; HWE: Hardy-Weinberg equilibrium;

^a^ Maternal blood

^b^ Cord blood
